# Quantum Information Remote Carnot Engines and Voltage Transformers

**DOI:** 10.3390/e21020127

**Published:** 2019-01-30

**Authors:** Jose Diazdelacruz, Miguel Angel Martin-Delgado

**Affiliations:** 1 Department of Applied Physics and Materials Engineering, Universidad Politecnica de Madrid, 28040 Madrid, Spain; 2 Department of Theoretical Physics, Universidad Complutense de Madrid, 28040 Madrid, Spain; 3 Center for Computational Simulation, Universidad Politecnica de Madrid, 28040 Madrid, Spain

**Keywords:** quantum information heat engines, quantum thermodynamics, entanglement entropy, quantum cryptography

## Abstract

A physical system out of thermal equilibrium is a resource for obtaining useful work when a heat bath at some temperature is available. Information Heat Engines are the devices which generalize the Szilard cylinders and make use of the celebrated Maxwell demons to this end. In this paper, we consider a thermo-chemical reservoir of electrons which can be exchanged for entropy and work. Qubits are used as messengers between electron reservoirs to implement long-range voltage transformers with neither electrical nor magnetic interactions between the primary and secondary circuits. When they are at different temperatures, the transformers work according to Carnot cycles. A generalization is carried out to consider an electrical network where quantum techniques can furnish additional security.

## 1. Introduction

**Figure 1:**
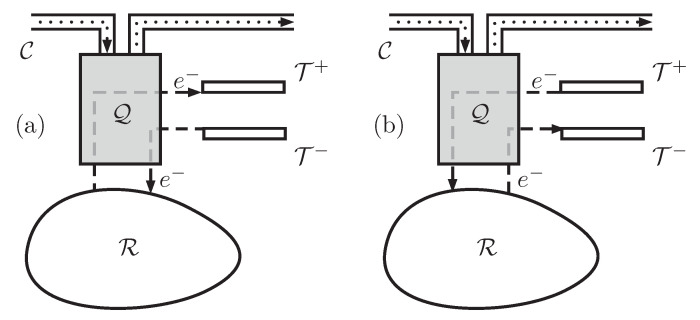
Simplified schematic representation of the function of a Quantum Information Electrical Generator (Quieg) Q. It features a communication line C through which qubits flow (dotted line). They enter Q in some state and exit at another one. A thermo-chemical reservoir R contains electrons at temperature *T* and chemical potential μ. They can be moved to or from two terminals $T^{+},T^{-}$ that also work as thermo-chemical reservoirs at temperature *T* and chemical potentials $V^{+},V^{-}$ (dashed line). Q may work in the direct (**a**) or reverse (**b**) mode. In the first case, the entropy of the qubits in C increases, whereas in the second one it decreases.

It is now well known that low-entropy qubits can be traded for useful work using an information heat engine and a thermal reservoir. In this paper, we describe a different kind of information heat engine that uses a thermo-chemical reservoir of electrons, instead of just a heat bath as in previous papers. The engine will be referred to as Quantum Information Electric Generator or Quieg. The system works in cycles and its net effect is two-fold: manipulating the entropy of a qubit going through a communication line and moving electrons between a thermo-chemical reservoir and two terminals at different electro-chemical potentials. By a suitable transfer of electrons, the system can either borrow electrostatic energy from the terminals or supply it to them. In the first case, the entropy of the qubits flowing in the line will increase. It is represented in Figure 1a and will be referred to as the direct mode of the Quieg. In the second case, the entropy of the qubits flowing in the line will decrease. It is represented in Figure 1b and will be referred to as the reverse mode. Conservation of the energy and the number of electrons is possible because there is an available reservoir with well-defined temperature and chemical potential for electrons. We describe a thermodynamic cycle where, after everything has been taken into consideration, the number of electrons extracted from the reservoir is proportional to the variation of the entropy of the qubits in the communication line. Section 3 contains a detailed description of the structure of a Quieg. Section 4 and Section 5 describe the different processes involved in the direct and reverse modes, respectively.

The number of qubits needed to transfer one electron is a dimensionless quantity that we define as info-potential in Section 4 and is related to temperature and chemical potential.

When two distant Quiegs share a communication line, one of them can use the qubits flowing through it to supply electricity to local consumers, while the other can reset the qubits at the expense of using up the charge stored in its terminals. This can be regarded as a voltage transformer, which uses qubits instead of a magnetic field as a mediator. Interestingly, there is neither upper nor lower bounds for the voltage of the terminals that receive or emit electrons. The system is analyzed in Section 6 and will be referred to as informational voltage transformer.

It will be shown that when the Quiegs are at different temperatures, the transformer becomes a variation of a Carnot engine, with the same efficiency. This is explored in Section 7. It will be referred to as informational Carnot engine.

One can then envisage an efficient electric energy network with consumers and suppliers placed at distant points with access to different thermo-chemical reservoirs and Quiegs. Neither energy nor electromagnetic fields, electrons or any other electrically charged particles will travel from node to node. Yet, the system will enable the distribution of power in the network. Charges flowing in electrical currents will be extracted from or stored in the local reservoirs.

A network policy is needed to share the communication line. It should grant access to the low-entropy qubits only for the right Quiegs. Quantum techniques are employed to render the information useless for anyone but the legitimate node. They are presented in Section 9.

Finally, Section 10 contains a discussion of the possible problems of the system. Some general conclusions are presented in Section 11.

## 2. Antecedents

It is widely accepted that the first information heat engine was introduced in 1871 by J.C. Maxwell in his Theory of Heat [1]. He considered the possibility of a microscopic demon which could obtain work after gaining information about the position and velocity of single particles. Long afterward, in 1929 L. Szilard proposed his celebrated engine [2]. These two early contributions were considered by many scientists as a challenge to the second principle of thermodynamics. More precisely, they seemed to contradict the statement regarding the impossibility of using the thermal energy contained in a single heat bath to cyclically fuel an engine whose output was work done on an external system. Arguably, the question was not definitely settled until many years later. Different contributions [3,4,5,6] have explained that there is no contradiction between the second law and Szilard’s engine or Maxwell’s demon. In our opinion, the main difficulty in tying the second law and engines lies in the presence of feedback loops [6,7]. Fundamental physical laws establish that the evolution of a system should occur according to some Hamiltonian. Hence, it must be reversible. However, many feedback loops work to steer a system towards a definite final state regardless of its initial situation. Careful analysis reveals that the entropy lost in the system must go elsewhere. Most feedback control systems need to cyclically dump some entropy into their environment. This interaction is somewhat elusive and is often absent in the analysis, what leads to contradictions with the second law.

Other situation where there is a need to get rid of entropy is resetting computers. This operation forgets the previous state of the memory; therefore, is irreversible and cannot be performed unless information about the memory goes out as entropy. In 1951, Landauer in a celebrated paper [8] linked this entropy to heat dissipation in a thermal bath, which was recently measured in 2012 [9]. Bennett [10] later established that any further step of computation may be reversible, provided that a suitable number of ancillary bits is available. The same principle applies to quantum computing [11,12,13,14]. Likewise, quantum sensors [15] also need a similar resetting step.

The laws of thermodynamics establish that entropy can be cyclically reset if there is a heat bath and some work is done on the system. Reversely, some work can be done by cyclically increasing the entropy of some ancillae. This is performed in information heat engines [4,5,16,17,18,19,20,21,22,23,24,25,26,27,28,29,30,31,32]. The states of the ancillae can be classical or quantum. Some papers [33,34,35,36] have elucidated the differences between the two.

The capacity of work production of an ancillary system in a definite state is related to its Kullback-Leibler divergence with respect to its thermal equilibrium state for classical systems and to the relative entropy for quantum ones [37,38,39,40]. They are equal to standard entropies when the thermal equilibrium state is distributed uniformly. This is the case of the qubits in the communication line presented in this paper. This assumption is equivalent to a degenerate Hamiltonian for the states of the qubit. Systems that store information which can be traded for work [41], hot reservoir refrigeration [42] or any other thermodynamically valuable function are called informational reservoirs. They are usually considered as a memory register on which information can be written or erased and some other valuable parameter is thereby produced or consumed. They provide new factors and analysis in stochastic thermodynamics, which has needed to be generalized to include informational reservoirs [43]. Correlations between qubits in a memory can also be exploited and furnish additional advantage [44]. A more generalized consideration of work extraction from quantum correlations can be found in Reference [45]. Smooth entropies have also been defined [46,47,48] for the case in which not only averages, but some other aspects of work production, as confidence intervals, etc. are considered. They focus on single shot rather than infinite copies thermodynamics.

Quantum Heat Engines [49,50] and Electronic Systems [51] have also been studied. They are devices that work with two local baths at different temperatures (or chemical potentials in Electronics) and follow the function of their classical counterparts. Carnot and Otto cycles have been particularly analyzed. Assuming thermodynamic conditions, they reach ideal values of efficiency. However, they would take infinite amounts of time to adiabatically finish some stages. To do it in finite time, some shortcuts to adiabaticity [52,53,54,55,56] have been proposed and analyzed. It leads to a trade-off between power and efficiency. In some situations, quantum supremacy over classical has been claimed and proved [57]. In this paper, we do not consider the infinite time limitation of adiabatic processes and assume that they are performed in a finite time with sufficient approximation.

Closely related to our system are some theoretical and experimental papers where electrons are transferred between thermo-chemical reservoirs under the presence of Maxwell’s demons. In Reference [58] electrons are moved against a potential using the entropy generation associated with information erasure in the detector. Reference [59] describes a theoretical model with an autonomous Maxwell’s demon coupled to a Single Electron Transistor (SET), which can extract entropy from the system and dump it into its own thermo-chemical reservoir. This kind of system has been demonstrated [60] in an experimental setup, where the action of the demon was revealed by measuring the cooling of a reservoir connected to the SET and the heating of a reservoir connected to the demon.

Resource theories [61] provide a framework to identify what quantum states are useful to achieve some goal, provided there is a set of free available operations and states. In this paper, part of the system, namely the M qubits that move through the communication line C, are supposed to be a resource for the operation of the Quiegs. Their free states are the maximally mixed ones and their free operations are all unitary transformations and tracing out any number of qubits. Therefore, our M qubits part fits into the Resource Theory of Noisy Operations [62]. The free states and operations just mentioned make sense in a thermodynamic environment only if the Hamiltonian for the M qubits is always degenerate and constant. We assume that this is a reasonable assumption for the M qubits (not for other parts of the Quiegs),whose only function is to carry information. This restriction could be lifted and then the adequate framework would be the Resource Theory of Thermal Operations or of Gibbs Preserving Maps [63,64,65].

There are also papers [66,67,68] dealing with the use of quantum information techniques to protect the energy of a system shared by remote users. Our approach differs in that they use a non-local system to store the energy, whereas our approach only considers interaction between them through the exchange of qubits.

## 3. Elements of a Quieg

**Figure 2:**
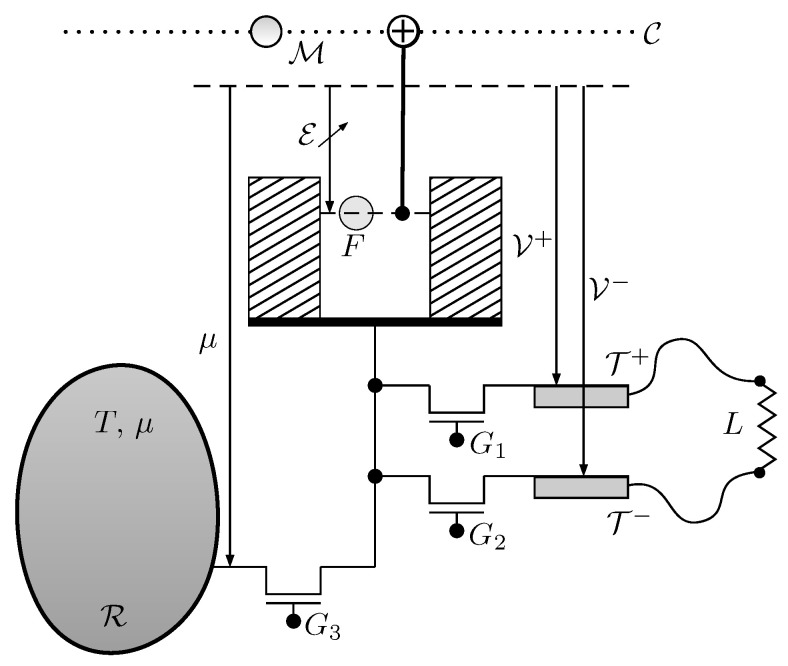
Elements of a Quieg. In the direct mode, qubits M travel through the communication line C. They are used as targets in a CNOT gate which is controlled by a fermionic system *F*. *F* features a tunable energy level E and can be either empty or filled with one single electron. After measuring M in the computational basis, *F* undergoes a suitable process whose outcome is either (1) extracting an electron from the thermo-chemical reservoir R at electro-chemical potential μ and deliver it to the negative terminal $T^{-}$ at voltage $V^{-}$ or (2) extracting an electron from the positive terminal $T^{+}$ at voltage $V^{+}$ and deliver it to the thermo-chemical reservoir R. Gates $G_{1},G_{2},G_{3}$ represent the possibilities of putting *F* in thermo-chemical equilibrium with $T^{+},T^{-},R$, respectively. *L* is an electrical load that draws electrons from $T^{-}$ at voltage $V^{-}$ and delivers them to $T^{+}$ at voltage $V^{+}$.

A Quieg has the capacity to increase the entropy of a qubit supplied by the communication line and transfer a suitable number of electrons from the local reservoir to the positive or negative local voltage terminals. It can also do the reverse process. Figure 2 depicts the structure of a Quieg. It contains:

1. A fermionic system *F* with only one available state. It can be either empty or occupied by a single electron.
2. A thermo-chemical reservoir R, at temperature *T* and electron chemical potential μ.
3. Two terminals $T^{+},T^{-}$ of a battery at potentials $V^{+},V^{-}$, respectively, used to power an electrical load *L* (direct mode) or to draw energy from an external source (reverse mode). They are equivalent to thermo-chemical reservoirs for electrons at temperature *T* and chemical potentials $V^{+},V^{-}$, respectively.
4. Three gates $G_{1},G_{2},G_{3}$ used to commute between the following four possible situations for *F*:
  i. it is isolated
  ii. it is in equilibrium with the terminal $T^{+}$
  iii. it is in equilibrium with the terminal $T^{-}$
  iv. it is in equilibrium with the reservoir R.
5. A tunable energy E for *F*.

**Figure 3:**
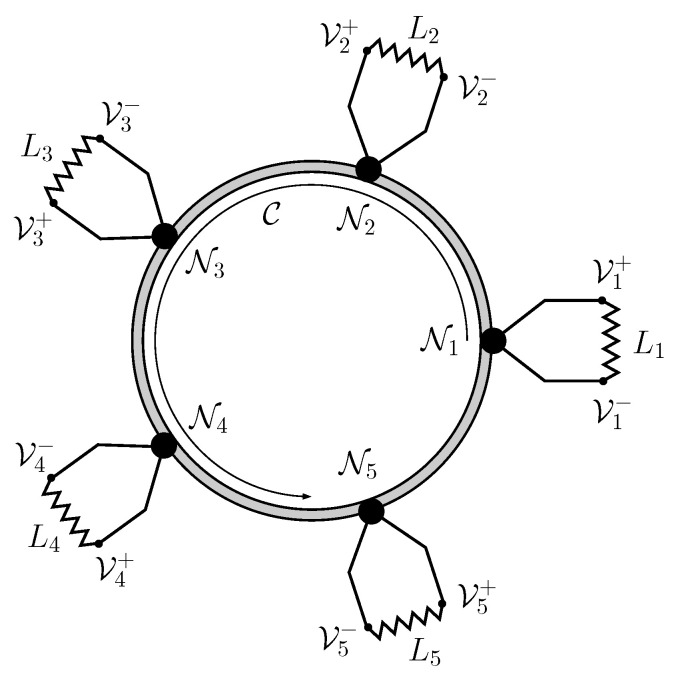
Nodes in a five-station ring-network. Each one should feature the elements represented in Figure 2. The communication line C contains qubit strings with an identification of source and destination ports. Each station can either order or disorder the qubits so that another station should reverse the operation. The net effect is that the nodes whose Quiegs work in the direct mode can deliver power to a passive load, whereas the nodes with Quiegs running in the reverse mode will act as power stations.

By a suitable cycling of control commands $G_{1},G_{2},G_{3}$, measurements of *F* in M and tuning of energy E, further sections will show that a set of remote stations (as those shown in Figure 3) can operate as an electrical network without actually interchanging any energy or electrons between them.

The physical implementation that we think of is based on a single electron quantum dot as was demonstrated for implementing a Carnot cycle [69,70]. The system can be isolated from the environment so that a quantum state can live for some microseconds without significant decoherence. It is also possible to equilibrate the dot with a thermo-chemical environment which can be selected through a proper switching of electronic field effect transistors. Finally, it can implement CNOT gates with external qubits, as is needed in the process described in Section 4.

## 4. Stages of a Quieg Working in the Direct Mode

The Quieg works cyclically. We will describe first the cycle corresponding to the case where the M qubits increase their entropy (Figure 1a). The initial state of M is a partially mixed state given by

(1) $$\rho_{\mathrm{init}}^{M}=m|0\rangle \langle 0|+\left(1-m\right)|1\rangle \langle 1|,$$

where $\frac{1}{2}\leq m\leq 1$. Please note that by redefinition of the $\left\{|0\rangle ,|1\rangle \right\}$ basis, any state of M can be cast in the format given by Equation (1). The fermionic system *F* is initially in equilibrium with reservoir R at an energy level $E_{0}=\mu$ such that *F* is maximally mixed. Accordingly, its initial state is

(2) $$\rho_{\mathrm{init}}^{F}=\frac{1}{2}|0\rangle \langle 0|+\frac{1}{2}|1\rangle \langle 1|.$$

Then, it will undergo the following stages:

1. *Measurement*. The qubit M from C acts as a target qubit of a CNOT gate controlled by *F*. The bipartite system MF results in the joint state
  (3) $$\rho_{CNOT}^{\left(M\;F\right)}=\frac{1}{2}\left(m|00\rangle \langle 00|+m|11\rangle \langle 11|+\left(1-m\right)|01\rangle \langle 01|+\left(1-m\right)|10\rangle \langle 10|\right).$$
  After measuring, M will be either in $|0\rangle$ or $|1\rangle$ state with equal probabilities. The states of *F*, conditioned to the outcomes, are
  (4) $$\left\{\begin{array}{ccc} \rho_{0}^{F} & = & m|0\rangle \langle 0|+\left(1-m\right)|1\rangle \langle 1|\\ \rho_{1}^{F} & = & m|1\rangle \langle 1|+\left(1-m\right)|0\rangle \langle 0|\;. \end{array}\right.$$
2. *Branching*. According to the value of M, the process may take two possible ways. First, we assume that *F* is in state $\rho_{1}^{F}$, where it is more likely to be occupied (case a) and then we describe the steps taken if *F* is in state $\rho_{0}^{F}$ (case b), when it is more likely to be empty.

If M is in the $|1\rangle$ state, the following steps are:

a.3. *Fitting*. The energy level E is tuned to $E_{1}$, while *F* is isolated. The value of $E_{1}$ is the one for which the equilibrium state, when *F* is in contact with $T^{+}$, is $\rho_{1}^{F}$. It is given by
  (5) $$m=\frac{1}{1+e^{\beta (E_{1}-V^{+})}}.$$
a.4. *Plunging*. *F* is put in contact with $T^{+}$ and, keeping the equilibrium, E is raised from $E_{1}$ to $E_{a}$, where there is a probability of occupation $p_{a}$ to be determined later. Accordingly, $E_{a}$ results from
  (6) $$p_{a}=\frac{1}{1+e^{\beta (E_{a}-V^{+})}}\;.$$
a.5. *Leveling*. *F* is isolated and E is taken from $E_{a}$ to $E_{2}$ chosen so that $p_{a}$ is the occupation probability for *F* at equilibrium with R. $E_{2}$ satisfies the equation
  (7) $$p_{a}=\frac{1}{1+e^{\beta (E_{2}-\mu )}}\;.$$
a.6. *Relaxation*. While in contact with R, E is taken back from $E_{2}$ to $E_{0}$ as it drives *F* back to its initial state $\rho_{\mathrm{init}}^{F}$.

For the case where M is in the $|0\rangle$ state, the process follows according to:

b.3. *Fitting*. The energy level E is tuned to $E_{1}^{\prime }$, while *F* is isolated. The value of $E_{1}^{\prime }$ is the one for which the equilibrium state when *F* is in contact with $T^{-}$ is $\rho_{0}^{F}$. It is given by
  (8) $$1-m=\frac{1}{1+e^{\beta (E_{1}^{\prime }-V^{-})}}\;.$$
b.4. *Plunging*. *F* is put in contact with $T^{-}$ and E is lowered from $E_{1}^{\prime }$ to $E_{b}$, where there is a probability of occupation $p_{b}$ to be determined later. Accordingly, $E_{b}$ results from
  (9) $$p_{b}=\frac{1}{1+e^{\beta (E_{b}-V^{-})}}\;.$$
b.5. *Leveling*. *F* is isolated and E is taken from $E_{b}$ to $E_{2}^{\prime }$ chosen so that $p_{b}$ is the occupation probability for *F* at equilibrium with R. It satisfies the equation
  (10) $$p_{b}=\frac{1}{1+e^{\beta (E_{2}^{\prime }-\mu )}}\;.$$
b.6. *Relaxation*. While in contact with R, E is taken back from $E_{2}^{\prime }$ to $E_{0}$ as it drives *F* back to its initial state $\rho_{\mathrm{init}}^{F}$.

**Figure 4:**
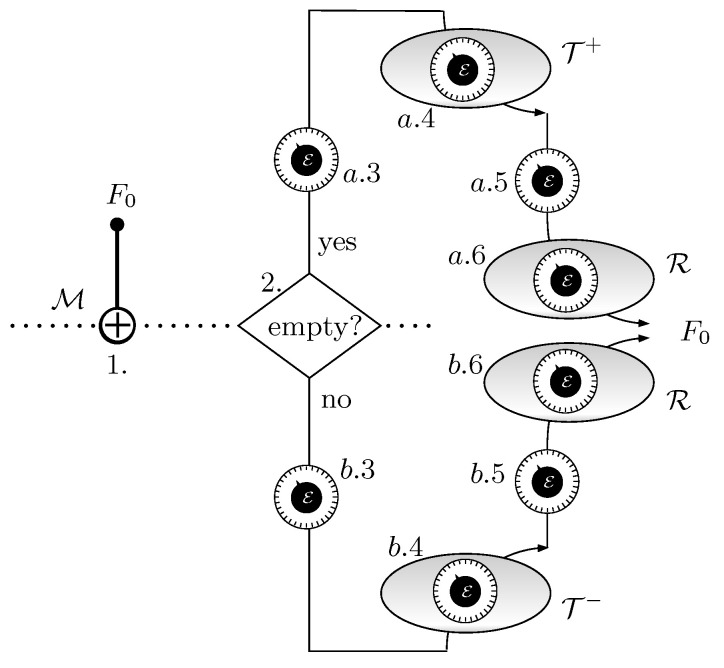
Symbolic diagram of the stages of the system. The clock-like elements represent tuning knobs for setting the adequate values of E. The shaded ovals indicate equilibrium with a thermo-chemical reservoir. First, *F* is measured by means of a CNOT gate and the result is placed at M. According to its value, the upper or the lower part of the graph is used. If M is 1, the energy E is lowered so that *F* can be reversibly put in contact with $T^{+}$. Then it is raised keeping *F* in equilibrium with $T^{+}$, until the probability of occupation reaches the value $p_{a}$. At this point, *F* is put in isolation and E is lowered to put *F* in equilibrium with reservoir R. Then, in contact with R, E is further raised until the initial state is reached. If M is 0, the energy E is raised so that *F* can be reversibly put in contact with $T^{-}$. Then it is lowered keeping *F* in equilibrium with $T^{-}$, until the probability of occupation reaches the value $p_{b}$. At this point, *F* is put in isolation and E is raised to put *F* in equilibrium with reservoir R. Then, in contact with R, E is further lowered until the initial state is reached.

**Figure 5:**
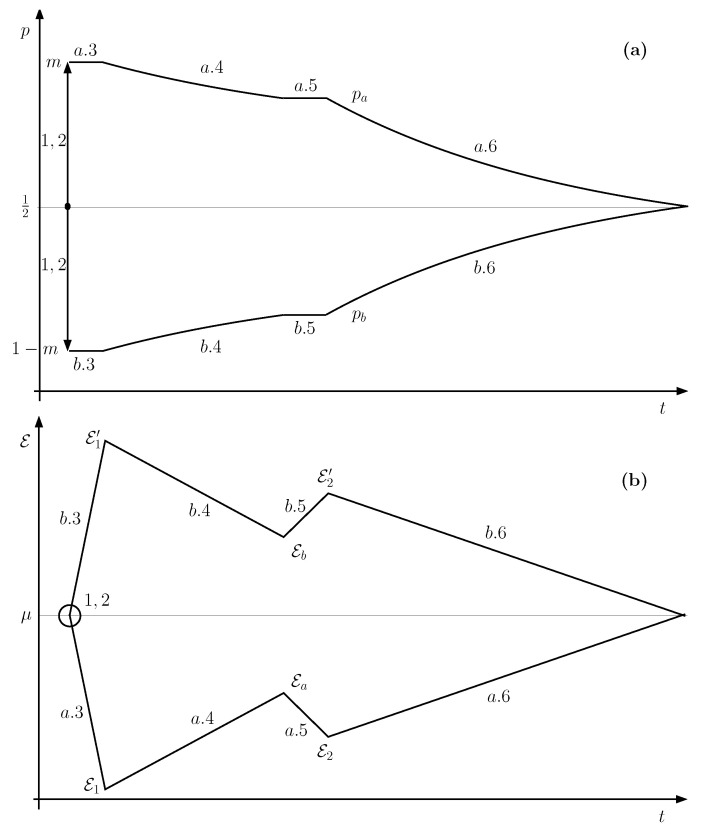
Evolution of the occupation probability *p* of *F* (**a**) and the tunable energy E; (**b**) for a Quieg working in the direct mode.

Figure 4 represents the whole schedule. Figure 5 contains the evolution of the occupation probability *p* of *F* and the tunable energy E. Considering that the probabilities of occupation for *F* at the beginning and the end of stage a.4 are $m,p_{a}$, respectively, the average number of electrons transferred per cycle from R to $T^{+}$ is

(11) $$\;\langle \;N_{R\to T^{+}}\;\rangle \;=\frac{1}{2}\left(m-p_{a}\right)\;.$$

Similarly, taking into account that the occupation probabilities for *F* at the beginning and the end of stage b.4 are 1−m and $p_{b}$, the average number of electrons transferred to R from $T^{-}$ is

(12) $$\;\langle \;N_{R\to T^{-}}\;\rangle \;=\frac{1}{2}\left(p_{b}-\left(1-m\right)\right)\;.$$

We require that they are equal, so that

(13) $$1-p_{a}=p_{b}\;.$$

Each of the listed stages except the *Measurement* is a reversible process. Therefore, the following equation holds

(14) $$d\;U\;=\;-d\;W\;+\;\left(\ln2\right)\;k_{B}\;T\;d\;S\;+\;\mu \;dp\;,$$

where U:=pE is the average energy of *F* and −dW is the differential work done in the cycle by the source of E on *F*. Measurement puts *F* in either a $m\;|0\rangle \langle 0|\;+\;\left(1-m\right)\;|1\rangle \langle 1|$ or $\left(1-m\right)\;|0\rangle \langle 0|\;+\;m\;|1\rangle \langle 1|$ states with equal probabilities; accordingly, the average value of *U* is not changed in this stage. This implies that

(15) $$\;\langle \;\int_{r}dU\;\rangle \;=\;\langle \;\oint dU\;\rangle \;=0\;,$$

where subscript *r* stands for integration over the reversible path, which is formed by the 3.a-4.a-5.a-6.a or 3.b-4.b-5.b-6.b stages. Equation (14) now yields

(16) $$-\;\;\langle \;\int_{r}\;d\;W\;\;\rangle \;+\;\left(\ln2\right)\;k_{B}\;\;\langle \;\int_{r}\;\left(\;T\;d\;S\;+\;\mu \;dp\right)\;\rangle \;\;=\;0\;.$$

The averages for the cases (a) and (b) are

(17) $$-W_{a}\;+\;\left(\ln2\right)\;k_{B}\;T\times \left(1-S_{m}\right)+\left(p_{a}-m\right)V^{+}+\left(\frac{1}{2}-p_{a}\right)\mu \;,$$

(18) $$-W_{b}\;+\;\left(\ln2\right)\;k_{B}\;T\times \left(1-S_{m}\right)+\left(p_{b}-1+m\right)V^{-}+\left(\frac{1}{2}-p_{b}\right)\mu \;,$$

where

(19) $$S_{m}:=-m\;\log_{2}m-\left(1-m\right)\;\log_{2}\left(1-m\right)$$

is the entropy of $\rho_{\mathrm{init}}^{M}$. The cases (a) and (b) occur with equal probabilities so that averaging Equation (17) and Equation (18) yields

(20) $$-\frac{W_{a}+W_{b}}{2}\;+\;\left(\ln2\right)\;k_{B}\;T\times \left(1-S_{m}\right)+\frac{1}{2}\;\left[\left(p_{a}-m\right)V^{+}+\left(p_{b}-1+m\right)V^{-}\right]+\frac{1}{2}\;\left(1-p_{a}-p_{b}\right)\mu =0\;.$$

Plugging Equation (13) into Equation (20) we arrive at

(21) $$-\frac{W_{a}+W_{b}}{2}\;+\;\left(\ln2\right)\;k_{B}\;T\times \left(1-S_{m}\right)+\frac{1}{2}\;\left(p_{a}-m\right)\left(V^{+}-V^{-}\right)=0\;,$$

whence the value of $p_{a}$ which makes the average work vanish results

(22) $$p_{a}=m-\frac{2\;\left(\ln2\right)\;k_{B}\;T\;\left(1-S_{m}\right)}{V^{+}-V^{-}}\;,$$

which, substituted in Equation (21), yields

(23) $$\frac{W_{a}+W_{b}}{2}=0\;.$$

Therefore, the average number of electrons $\;\langle \;N_{T^{-}\;\to \;T^{+}}\;\rangle \;$ transferred from terminal $T^{-}$ to terminal $T^{+}$ per cycle is

(24) $$\;\langle \;N_{T^{-}\;\to \;T^{+}}\;\rangle \;=\frac{\left(\ln2\right)\;k_{B}\;T\;\left(1-S_{m}\right)}{V^{+}-V^{-}}\;.$$

Accordingly, the average number $\;\langle \;M_{T^{-}\;\to \;T^{+}}\;\rangle \;$ of qubits M needed per electron transferred is

(25) $$\;\langle \;M_{T^{-}\;\to \;T^{+}}\;\rangle \;=\frac{V^{+}-V^{-}}{\left(\ln2\right)\;k_{B}\;T\;\left(1-S_{m}\right)}\;.$$

The entropy of the qubit M is increased from $S_{m}$ to 1 in every cycle. This prompts us to define a new kind of dimensionless potential $\gamma_{R}$ for any thermo-chemical reservoir of electrons, which we call info-potential (this quantity is closely related to the logarithm of the fugacity) and is given by

(26) $$\gamma_{R}:=\frac{\mu }{\left(\ln2\right)\;k_{B}\;T}\;,$$

which represents the number of pure state qubits required per electron transferred to the reservoir $R_{n}$ from a zero chemical potential reservoir. Alternatively, it can be envisaged as the number of bits per particle by which the entropy of M must increase. It can be referred to using the dimensionless expression: bits per particle.

Terminals $T^{+},T^{-}$ also feature info-potentials $\gamma^{+},\gamma^{-}$, given by

(27) $$\left\{\begin{array}{ccc} \gamma^{+} & := & \frac{V^{+}}{\left(\ln2\right)\;k_{B}\;T}\\ \gamma^{-} & := & \frac{V^{-}}{\left(\ln2\right)\;k_{B}\;T}\;. \end{array}\right.$$

Using them, Equation (24) can be written as

(28) $$\;\langle \;N_{T^{-}\;\to \;T^{+}}\;\rangle \;=\frac{\left(1-S_{m}\right)}{\gamma^{+}-\gamma^{-}}\;.$$

Equations (23) and (24) establish that when a Quieg acts as a particle source, the information of the qubits in C is converted into particles accumulated at the terminals.

## 5. Quieg Working in the Reverse Mode

**Figure 6:**
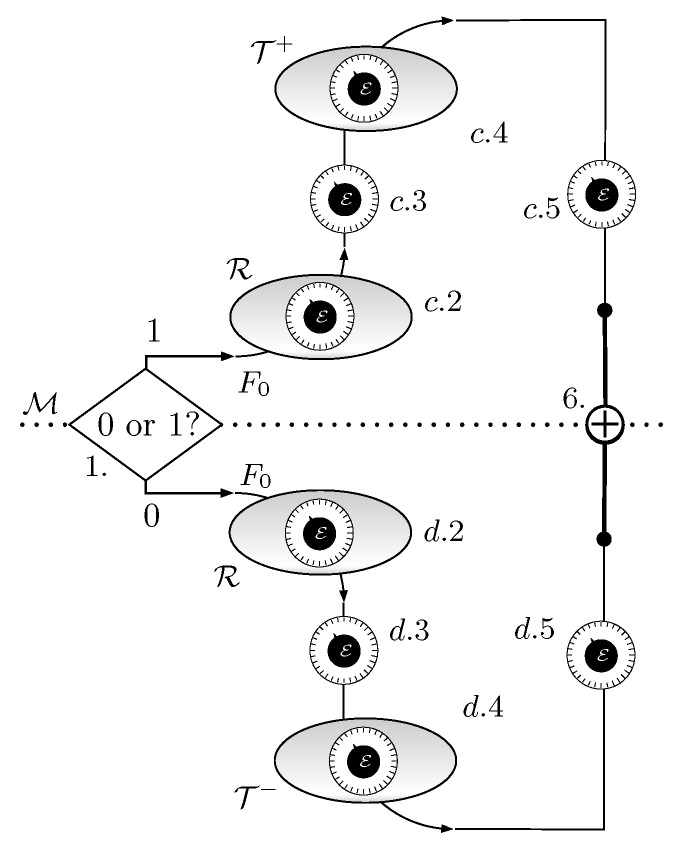
Stages of the Quieg working in the reverse mode. First, M is measured. According to the result, the system follows the upper branch or the lower one. If the measurement determines that M is in state $|1\rangle$, then steps a.6, a.5, a.4, a.3 are reversed and *F* is left in state $m\;|0\rangle \langle 0|\;+\;\left(1-m\right)\;|1\rangle \langle 1|$. If the measurement determines that M is in state $|0\rangle$, then steps b.6, b.5, b.4, b.3 are reversed and *F* is left in state $\left(1-m\right)\;|0\rangle \langle 0|\;+\;m\;|1\rangle \langle 1|$. A CNOT controlled by *F* and targeted at M factorizes the system in a maximally mixed state for *F* and a $m\;|0\rangle \langle 0|\;+\;\left(1-m\right)\;|1\rangle \langle 1|$ state for M.

The Quieg described in Section 4 increases the entropy of the messenger qubits and transfers electrons from a lower energy to a higher energy terminal. In this section, we define a different cycle to do the inverse operation. The starting point is an *F* system in state $\rho_{\mathrm{init}}^{F}$, with the energy E set to $E_{0}=\mu$. The qubit M is in a maximally mixed state. Then the Quieg works according to the following stages:

1. *Branching*. The qubit M is projected into its computational basis and one of two paths is selected. The value 1 (case c) branches into a sequence of steps which correspond to the upper part of Figure 6, whereas the value 0 (case d) selects the lower part of the figure.

Next, we describe the path corresponding to case c:

c.2. *Polarization*. It is the inverse of stage a.6. of Section 4. Initially, the system *F* is in equilibrium with reservoir R. The energy E is taken from $E_{0}$ to $E_{2}$, as defined in Equation (7) and the state of *F* is driven from $\rho_{\mathrm{init}}^{F}$ to $\rho_{a}^{F}$, given by
  (29) $$\rho_{a}^{F}=p_{a}|0\rangle \langle 0|+\left(1-p_{a}\right)|1\rangle \langle 1|\;,$$
  where $p_{a}$ is defined in Equation (22).
c.3. *Adjustment*. In this stage, E is tuned from $E_{2}$ to $E_{a}$ as given by Equation (6) and is the inverse of a.5. *F* stays isolated and its state remains $\rho_{1}^{F}$.
c.4. *Extraction*. While *F* is in equilibrium with $T^{+}$, E is lowered from $E_{a}$ to $E_{1}$, so that the probability of occupation for *F* increases. Its state is
  (30) $$\rho_{m}^{F}=m|0\rangle \langle 0|+\left(1-m\right)|1\rangle \langle 1|\;.$$
  This stage is the inverse of a.4.
c.5. *Zeroing*. This stage adiabatically takes the energy E back to $E_{0}$.

In the case d:

d.2. *Polarization*. This corresponds to the inverse of stage b.6. of Section 4. The system *F* is in equilibrium with reservoir R. The energy E is taken from $E_{0}$ to $E_{2}^{\prime }$, as defined in Equation (10) and the state of *F* is driven from $\rho_{\mathrm{init}}^{F}$ to
  (31) $$\rho_{b}^{F}=p_{b}|0\rangle \langle 0|+\left(1-p_{b}\right)|1\rangle \langle 1|\;.$$
d.3. *Adjustment*. In this stage, E is tuned from $E_{2}^{\prime }$ to $E_{b}$ as given by Equation (9) and is the inverse of b.5. *F* stays isolated.
d.4. *Extraction*. While *F* is in equilibrium with $T^{-}$, E is lowered from $E_{b}$ to $E_{1}^{\prime }$, so that the probability of occupation for *F* decreases. This stage is the inverse of b.4.
d.5. *Zeroing*. This stage adiabatically takes the energy E back to $E_{0}$.

A final stage is applied to both branches.

**Figure 7:**
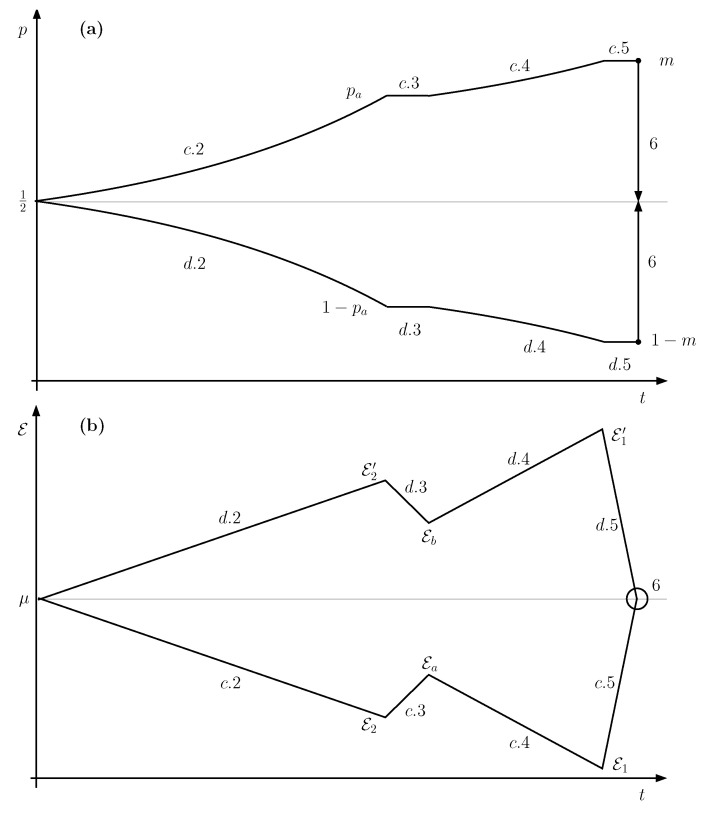
Evolution of the occupation probability *p* of *F* (**a**) and the tunable energy E; (**b**) for a Quieg working in the reverse mode.

6. *Reset*. The final step of the cycle is a CNOT gate controlled by *F* targeted at M. The state of the M−F system before applying the gate is
  $$\rho_{5}^{\left(M-F\right)}=\frac{1}{2}m\left(|00\rangle \langle 00|+|11\rangle \langle 11|\right)+\frac{1}{2}\left(1-m\right)\;\left(|01\rangle \langle 01|+|10\rangle \langle 10|\right)\;.$$
  In addition, after the CNOT, is
  $$\rho_{6}^{\left(M-F\right)}=\frac{1}{2}m\left(|00\rangle \langle 00|+|01\rangle \langle 01|\right)+\frac{1}{2}\left(1-m\right)\;\left(|11\rangle \langle 11|+|10\rangle \langle 10|\right)\;,$$
  which factorizes into
  $$\rho_{6}^{\left(M-F\right)}=\left[m|0\rangle \langle 0|+\left(1-m\right)|1\rangle \langle 1|\right]\;⊗\;\frac{1}{2}\left(|0\rangle \langle 0|+|1\rangle \langle 1|\right)\;.$$
  Therefore, it leaves *F* in a maximally mixed state and M in state $\rho_{\mathrm{init}}^{M}$ given by Equation (1).
  Figure 6 represents the whole schedule. Figure 7 contains the evolution of the occupation probability *p* of *F* and the tunable energy E.
  The energy balance follows that of Section 4. The only difference is that the processes are reversed. Accordingly, the number of M qubits generated per electron transfer from $T^{+}$ to $T^{-}$ is
  (32) $$\;\langle \;N_{T^{+}\;\to \;T^{-}}\;\rangle \;=\frac{\left(1-S_{m}\right)}{\gamma^{+}-\gamma^{-}}\;.$$

## 6. Informational Voltage Transformer

In Section 4 and Section 5 two ways of working for Quiegs have been described. A Quieg working in the direct mode supplies electrons to a higher energy $V^{+}$ (lower voltage $V^{+}=-V^{+}/e$, −e is the electron charge) terminal and borrows them from a lower energy $V^{-}$ (higher voltage $V^{-}=-V^{-}/e$) terminal. The voltage difference $\Delta V:=V^{-}-V^{+}$ is related to the adjustment parameter $p_{a}$ through Equation (22). If a rate of qubits $B_{1}$ is available, it can supply a current to a load connected to a Quieg $Q_{1}$ according to the equation

(33) $$I_{1}=-eB_{1}\frac{1-S_{m}}{\gamma_{1}^{+}-\gamma_{1}^{-}}=-B_{1}\frac{\left(1-S_{m}\right)\left(\ln2\right)\;k_{B}\;T}{\Delta V_{1}}\;,$$

which leads to a power transfer given by

(34) $$P_{1}=-B_{1}\frac{\left(1-S_{m}\right)\left(\ln2\right)\;k_{B}\;T}{\Delta V}\left(V_{1}^{+}-V_{1}^{-}\right)=B_{1}\left(1-S_{m}\right)\left(\ln2\right)\;k_{B}\;T\;.$$

The qubits can be supplied by a second Quieg $Q_{2}$ working in the reverse mode under the setting

(35) $$p_{a}^{\prime }=m-\frac{2\;\left(\ln2\right)\;k_{B}\;T\;\left(1-S_{m}\right)}{e\;\Delta V_{2}}\;,$$

where $\Delta V_{2}=\frac{V_{2}^{-}-V_{2}^{+}}{e}$ is the potential difference at the terminals of $Q_{2}$. It draws power from them and uses it to deliver fresh qubits to the communication line C. The power delivered depends on the rate of qubits $B_{2}$ through the equation

(36) $$P_{2}=B_{2}\left(1-S_{m}\right)\left(\ln2\right)\;k_{B}\;T\;.$$

**Figure 8:**
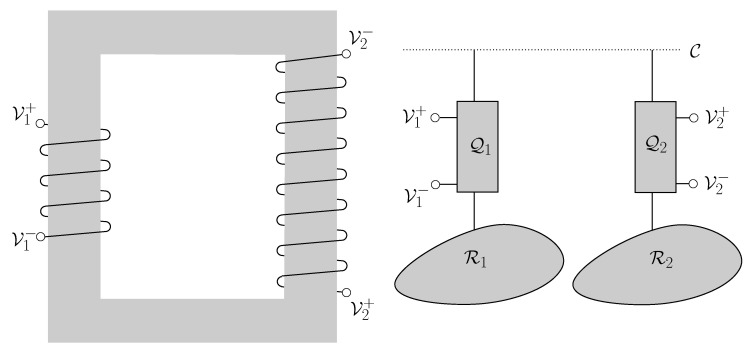
The **left** part of the figure represents a traditional voltage transformer. Magnetic coupling through a ferromagnetic core makes two electrical circuits exchange energy. The relation between the voltages is given by the ratio of the windings. In the **right** system, the coupling is mediated by a communication line C. Two nodes, each one with a Quieg share the qubits that travel through C. By setting the $p_{a}$ parameter, the voltage difference at each node is selected.

If $Q_{1}$ uses up all the qubits delivered by $Q_{2}$ then $B_{1}=B_{2}$ and $P_{1}=P_{2}$, as in conventional transformers (see Figure 8). Power is obtained from a voltage difference $\Delta V_{2}$ and delivered at $\Delta V_{1}$. Both potential differences can be adjusted by tuning the parameters $p_{a},p_{a}^{\prime }$, respectively.

## 7. Remote Informational Carnot Engine

Another interesting application of Quiegs is as a remote Carnot engine, when a Quieg is at a temperature $T_{1}$ and another one is at temperature $T_{2}<T_{1}$. We assume that the temperature of a Quieg is shared by its reservoir and its terminals. If we apply the analysis developed in Section 6 to the situation where the temperatures of $Q_{1}$ and $Q_{2}$ are different, we obtain the same relations for power $P_{1},P_{2}$ as in a traditional Carnot cycle.

**Figure 9:**
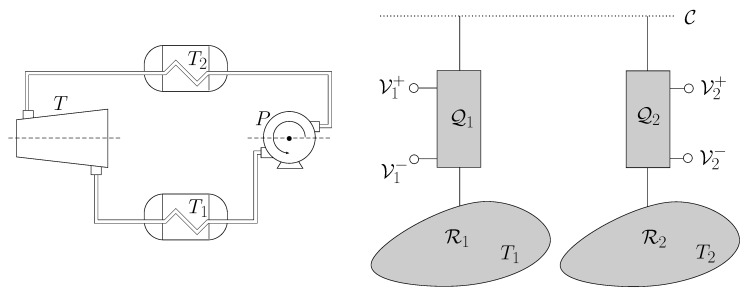
When the temperatures at the two nodes of an informational voltage transformer are different, the electrical energy supplied at one end may be bigger than the one consumed at the other. In fact, energy is drawn from the temperature difference. This follows immediately if we consider the temperature as the work cost of resetting a maximally mixed qubit which is the same as the work obtained by completely mixing a pure state qubit (within some multiplicative factors).

Using the expressions obtained in Equations (34) and (36), we obtain

(37) $$P_{1}=B_{1}\left(1-S_{m}\right)\left(\ln2\right)\;k_{B}\;T_{1}\;,$$

(38) $$P_{2}=B_{2}\left(1-S_{m}\right)\left(\ln2\right)\;k_{B}\;T_{2}\;,$$

and, assuming that all the qubits prepared by $Q_{2}$ are used by $Q_{1}$, we arrive at

(39) $$\frac{P_{1}}{P_{2}}=\frac{T_{1}}{T_{2}}\;,$$

so that the power injected in a low-temperature Quieg is amplified at the output of another high-temperature Quieg. The same relation holds for the works injected in the pump and output at a turbine of a classical Carnot cycle (Figure 9).

Please note that the only link between the hot and cold reservoirs in a Quieg Carnot cycle is a communication line, so that there is no need for isolation for pipes through which fluids at different temperatures flow. This reason makes Quiegs remarkable candidates for remote Carnot cycles.

## 8. Network

By sharing the communication line C, the nodes described in Section 3 make up a network whose purpose is to enable the generation, consumption and storage of electrical energy/charge.

Generating nodes put the qubits circulating over C in a $\rho_{\mathrm{init}}^{M}$ quantum state. Consuming nodes leave the qubits in a maximally mixed state. At any node, the number of qubits required per electron transfer is given by the info-potential difference between the terminals of the Quieg, which is set by its parameter $p_{a}$. The energy is proportional to the temperature of the Quieg.

The system thus defined acts as an informational power network which has the remarkable feature of implementing Carnot cycles whenever two nodes have different temperatures.

We assume that the system works with large sets of qubits with a small overhead containing identification of destination, origin, prize, *m*-value, type and length. When there is access of untrusted users, then quantum security techniques can be used. They are considered briefly in Section 9.

## 9. Security

In this section, we define a scenario where a node *A* generates some informational qubits M in a certain state and they should be used only by a node *B* to transfer electrons. To render the qubits useless to an illegitimate node *E*, the state of M should be maximally mixed for *E*. There are several ways to achieve this goal.

**Figure 10:**
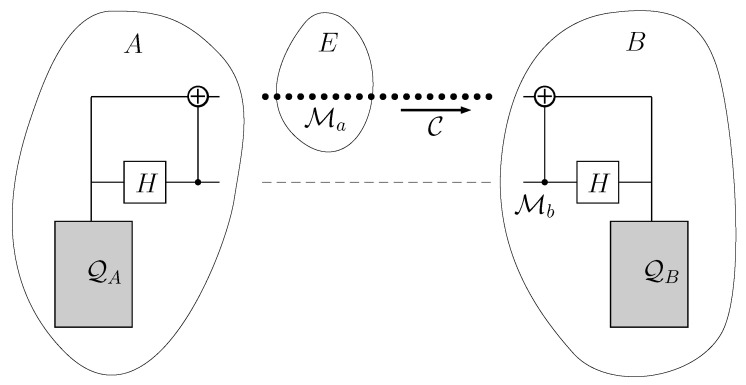
Outline of the security based on shared entangled qubit pairs. The Quieg $Q_{A}$ works in the reverse mode, outputting qubits in a $\rho_{\mathrm{init}}^{M}$ state. $M_{a},M_{b}$ are two of them. After they are generated, $M_{b}$ undergoes a Hadamard gate and controls a CNOT targeted at $M_{a}.$. Then, $M_{b}$ is taken to node *B*. This transfer is made before the system begins to work and may be done for a big number of $M_{a},M_{b}$ pairs. For example, it may take place before *B* signs-in into the network or when it renovates its subscription. It does not occur over the communication line C. Then, $M_{a}$ qubits circulate over C. When *B* needs to use them at $Q_{B}$, undoes the transformations performed by the CNOT and Hadamard gates. Then, *B* obtains two qubits in a $\rho_{\mathrm{init}}^{M}$ ready to be used in the $Q_{b}$ Quieg. Please note that if user *E* want to use $M_{a}$, it will be useless for a Quieg working in the direct mode, because it is in a maximally mixed state.

1. First, we consider a generalization of the technique explained in our previous paper [71]. It is depicted in Figure 10. *A* and *B* can share a set of entangled qubit pairs, all of them in the same state, upon which they have previously agreed. Their Quiegs are set to work with a set of qubits in the state $\rho_{\mathrm{init}}^{M}$ given by Equation (1). Accordingly, *A* uses qubits in a maximally mixed state and generates qubits in a $\rho_{\mathrm{init}}^{M}$ state, according to the reverse mode of the Quieg.
  Next, *A* applies an entangling unitary operation to a pair of qubits $M_{a},M_{b}$. First, a Hadamard gate is applied to $M_{b}$, so that it transforms into the state
  (40) $$\begin{array}{cc} \rho_{2}^{M_{b}} & =\frac{m}{2}\;\left(|0\rangle +|1\rangle \right)\;\left(\langle 0|+\langle 1|\right)+\frac{1-m}{2}\;\left(|0\rangle -|1\rangle \right)\;\left(\langle 0|-\langle 1|\right)\\ & =\frac{1}{2}\left(|0\rangle \langle 0|+|1\rangle \langle 1|\right)+\left(m-\frac{1}{2}\right)\;\left(|0\rangle \langle 1|+|1\rangle \langle 0|\right)\;. \end{array}$$
  Then, a CNOT controlled by $M_{b}$ takes the system from the joint state
  (41) $$\begin{array}{cc} \rho_{2}^{M_{a}M_{b}} & =\left(m\;|0\rangle \langle 0|+\left(1-m\right)\;|1\rangle \langle 1|\right)⊗\frac{1}{2}\left(|0\rangle \langle 0|+|1\rangle \langle 1|+\left(2m-1\right)\left[|0\rangle \langle 1|+|1\rangle \langle 0|\right]\right)\\ & =\frac{1}{2}\left(m\left[|00\rangle \langle 00|+|01\rangle \langle 01|\right]+m\left(2m-1\right)\left[|00\rangle \langle 01|+|01\rangle \langle 00|\right]\right)+\\ & \;\frac{1}{2}\left(\left(1-m\right)\left[|10\rangle \langle 10|+|11\rangle \langle 11|\right]+\left(1-m\right)\;\left(2m-1\right)\left[|10\rangle \langle 11|+|11\rangle \langle 10|\right]\right)\;, \end{array}$$
  to
  (42) $$\begin{array}{cc} \rho_{3}^{M_{a}M_{b}}= & \frac{1}{2}\left(m\left[|00\rangle \langle 00|+|11\rangle \langle 11|\right]+m\left(2m-1\right)\left[|00\rangle \langle 11|+|11\rangle \langle 00|\right]\right)+\\ & \frac{1}{2}\left(\left(1-m\right)\left[|10\rangle \langle 10|+|01\rangle \langle 01|\right]+\left(1-m\right)\;\left(2m-1\right)\left[|10\rangle \langle 01|+|01\rangle \langle 10|\right]\right)\;. \end{array}$$
  Next, *B* takes the $M_{b}$ part of the pair. The reduced states for $M_{a}$ and $M_{b}$ are equal. They are the partial traces of the matrix representing the state given by Equation (42) with respect to $M_{b}$ and $M_{a}$, respectively. They are given by
  (43) $$\rho_{3}^{M_{a}}=\frac{1}{2}\left(|0\rangle \langle 0|+|1\rangle \langle 1|\right)\;,$$
  which is the maximally mixed state. This implies that individually, $M_{a},M_{b}$ are useless. *B* keeps $M_{b}$ and *A* sends $M_{a}$ over the communication line C.
  Then, when *B* needs to fuel its Quieg working in the direct mode, takes $M_{a}$, so that it has the system $M_{a},M_{b}$ in the state $\rho_{3}^{M_{a}M_{b}}$ given by Equation (42). Subsequently, *B* applies a CNOT gate controlled by $M_{b}$ and targeted at $M_{a}$ followed by a Hadamard on $M_{b}$. This takes the pair to the state given by
  (44) $$\rho_{4}^{M_{a}M_{a}}:=\;\left(m|0\rangle \langle 0|+\left(1-m\right)|1\rangle \langle 1|\right)⊗\left(m|0\rangle \langle 0|+\left(1-m\right)|1\rangle \langle 1|\right)\;.$$
  This represents two M qubits in the $\rho_{\mathrm{init}}^{M}$ state, with which the Quieg at *B* can execute two cycles. So that the net effect is equivalent to the remote informational voltage transformer of Section 6 or a Carnot engine of Section 7. The only qubit traveling through the line is $M_{a}$ whose reduced state is maximally mixed and, therefore, useless for any unauthorized user *E*.
2. If *A* and *B* have no previous entangled states or secret key, then they must obtain a private key communicating through a public line. The classical RSA protocol can be used, but quantum computers could crack the key. On the other hand, QKD protocols could be used that offer quantum privacy. However, QKD consumes resources, and this implies that there is an additional cost for security. There have been several QKD protocols described. The best-known one is the BB84 [72], after Bennet and Brassard, who proposed it in 1984. These protocols consume resources because at some point they generate entropy. To reset the system for another cycle, the entropy must be evacuated elsewhere and this needs qubits in a not maximally mixed state (for example, the BB84 generates entropy when Bob measures the qubits sent by Alice in the wrong basis).

## 10. Discussion

The network described in Section 8 can be used to provide a way of distributing some resources between a set of generating and consumer nodes. These resources may be information, particle extraction or energy. Even though the idea may seem appealing, there are some potentially controversial points that we will address in this section.

It could be argued that the Quieg working in the direct mode, as presented in this paper, is equivalent a Quantum Information Heat Engine with a heat bath. However, the Quieg features a thermo-chemical reservoir of electrons, instead of just a heat bath. Accordingly, the Quieg evolves according to open system thermodynamics. It can absorb or release entropy, work and particles. The setting of $p_{a},p_{b}$ according to Equation (22) guarantees that average work is neither absorbed nor released. The Quieg trades bits for particles. In the case considered, the particles are electrons and their chemical potentials are the electrical potentials times the electron charge. Then, the electrons stored in a reservoir can serve different purposes. They can be used in a variety of situations, from causing an electrolytic chemical reaction to supply work to an electrical appliance or any other one. There is a direct relation between the electrons stored at two reservoirs at different potentials and the electrical work that can be obtained. From this point of view, the Quieg could be considered a Quantum Information Heat Engine. We think that it is not completely accurate because the production of electrical work is something that occurs outside the Quieg, whose only output is the displacement of electrons between the terminals.

Equation (22) sets a lower bound $\Delta^{*}$ for the ΔV that can be achieved for a given *m* value. It is expressed by

(45) $$\Delta V>\Delta^{*}=\frac{2\;\left(\ln2\right)\;k_{B}\;T}{em}\;\left(1-S_{m}\right)\;.$$

However, this restriction can be overcome by increasing $S_{m}$. It can be taken as close to 0 as necessary by making *m* approach $\frac{1}{2}$. Notably, there is no upper bound for the voltage.

We can estimate how much power can be distributed using today’s technology. Assuming T=300 K, $S_{m}=0$ and $B\approx 10^{12}$ bits/s, the power distributed is approximately

(46) $$P_{L}=\;B\;\left(\ln2\right)\;k_{B}\;T\;\approx 2,37\;\mathrm{nW}.$$

This value is very small when it is compared to the power managed by conventional electrical networks.

Another point is the necessity of using qubits instead of classical bits. Actually, the states of *F* and M are always classical mixtures of the $|0\rangle ,|1\rangle$ states. The only point where quantum systems are required are those related to the security of the network as described in Section 9. Classical bits are much easier to manipulate and send. This suggests that if there is no security concern, C can be a standard classical communication line. However, even in this case, the quantum treatment offers some advantages.

The entropy of the M qubits can be traded for work or particles at a cost which depends on the reservoir being used. The value of a qubit in work is given by $\left(\ln2\right)\;k_{B}\;T$ and in electron extraction capacity by γ. It is thus the entropy the adequate quantity whose value can be used as a kind of currency which can be exchanged for work or particles at a rate which depends on the node. Carnot cycles can be envisaged as a continuous flow of qubits between Quiegs which extracts a network because the work per bit at a node is different to its value at another node.

The way to obtain security is based on quantum information techniques. If a couple of entangled qubits is shared between producer and consumer the illegitimate user, has no possibility to obtain any benefit from accessing to an unauthorized qubit. However, a previous procedure must have ensured that the right users share the entangled qubits. If it is not possible, then a Quantum Key Distribution method can provide the security, at a further price of fresh qubits that must be used.

## 11. Conclusions

Qubits in a low-entropy state can be used as a resource for different purposes. The best-known one is the coding of messages to send or store information. However, there are other potential uses. If a heat bath is available, there is a wide variety of Quantum Information Heat Engines that can obtain useful work from the bath. Energy is distributed in a heat bath according to a maximum entropy principle. So is the number of particles in thermodynamic open systems. This paper shows that a Quieg can use the low-entropy qubits to move particles between reservoirs. In fact, it works like a Maxwell demon whose aim is to order particles, as opposed to energy. The Quieg is defined for a specific kind of particles: electrons. However, the system may work without significant changes for any other kind of fermion.

The use of two Quiegs in informational voltage transformers, as described in Section 6, represents the simplest network and, remarkably, just by choosing the $p_{a}$ parameter, may produce any voltage conversion. According to Section 7, when the temperatures of the Quiegs are different, the average value of the product of voltage times the electric current intensity is not the same in both nodes. There is an electrical power multiplication as in conventional Carnot cycles. More complex networks can be designed.

Even though the power or the number of particles that can be moved per qubit is low, it can be used in small electronic devices with reduced energy consumption. In fact, it can be used to supply Landauer’s resetting energy in quantum computing systems. Besides, the Quieg is inspired by quantum dots produced using semiconductor heterostructures.

Quantum techniques can guarantee privacy in quantum communication. It can also prevent unauthorized users to take advantage of the potential uses of qubits mentioned in the previous paragraph. However, this does not come without a cost. QKD procedures demand the use of more low-entropy qubits.
